# Left ventricular global function index by magnetic resonance imaging — a novel marker for differentiating cardiac amyloidosis from hypertrophic cardiomyopathy

**DOI:** 10.1038/s41598-020-61608-9

**Published:** 2020-03-13

**Authors:** Shan Huang, Hua-yan Xu, Kai-yue Diao, Ke Shi, Yong He, Sen He, Yi Zhang, Yue Gao, Meng-ting Shen, Ying-kun Guo, Zhi-gang Yang

**Affiliations:** 1 Department of Radiology, West China Hospital, Sichuan University, 37# Guo Xue Xiang, Chengdu, Sichuan, 610041 China; 2 Key Laboratory of Obstetric & Gynecologic and Pediatric Diseases and Birth Defects of Ministry of Education, National Key Laboratory of Biotherapy, West China Second University Hospital, Sichuan University, 20# South Renmin Road, Chengdu, 610041 China; 3 Department of Cardiology, West China Hospital, Sichuan University, 37# Guo Xue Xiang, Chengdu, 610041 China

**Keywords:** Magnetic resonance imaging, Cardiac hypertrophy

## Abstract

Differentiating cardiac amyloidosis (CA) from hypertrophic cardiomyopathy (HCM) remains a clinical challenge, particularly in those with preserved left ventricular ejection fraction (LVEF) and similar hypertrophy. This study aimed to use left ventricular global function index (LVGFI) and myocardial contraction fraction (MCF) to discriminate CA from HCM without using contrast agents on cardiovascular magnetic resonance imaging (CMR). In total, we included 68 CA patients, 90 HCM patients, and 35 healthy controls. We found that LVGFI had excellent diagnostic performance in differentiating CA from HCM (area under the curve (AUC) = 0.91, 95% CI [0.86–0.95]), even in the challenging conditions of similar hypertrophy (AUC = 0.92, 95% CI [0.87–0.97]) and preserved LVEF (AUC = 0.90, 95% CI [0.84–0.96]). LVGFI also had significant correlations with LGE extent, NT-proBNP and troponin T (all *p* < 0.001). Multiple logistic regression analysis revealed that LVGFI was an independent predictor of CA (odds ratio: 1.11, 95% CI: 1.01–1.23; *p* = 0.034). In conclusion, LVGFI is a novel and clinically useful parameters with excellent ability in determining myocardial function and differentiating cardiac amyloidosis from hypertrophic cardiomyopathy.

## Introduction

Cardiac amyloidosis (CA) is a progressive and infiltrative cardiomyopathy with poor prognosis. Left ventricular hypertrophy (LVH) is a typical sign of CA^1^. Differentiating CA from LVH caused by other etiologies such as hypertrophic cardiomyopathy (HCM) remains a clinical challenge, particularly in those patients with preserved left ventricular ejection fraction (LVEF) and similar hypertrophy. Early diagnosis of CA is critical for the selection of appropriate therapy and improvement of prognosis^2,3^.

At present, late gadolinium enhancement (LGE) of cardiovascular magnetic resonance imaging (CMR) is of great diagnostic value in diagnosing CA in clinical settings^4,5^. However, CA patients frequently suffer from renal failure, either due to amyloid deposition in the kidneys^6^ or due to reduced cardiac output from heart failure (HF), which makes them contraindicated to contrast agents^7^. In addition, early-stage CA may not present with typical LGE patterns. Thus, using a non-contrast method for diagnosing CA and avoiding the contrast-induced renal injury seem extremely important.

Whereas conventional non-contrast index LVEF is often preserved or ‘normal’ in patients with myocardial hypertrophy and HF symptoms^8^; therefore, it is not an optimal marker for the assessment and discrimination of CA from other types of hypertrophy^9^. Although LVEF may be remarkably high in some hypertrophic patients, they may have smaller LV volumes result from thickened walls and their higher LVEF does not necessarily produce sufficient cardiac output^10^.

Left ventricular global index (LVGFI) and myocardial contraction fraction (MCF), initially introduced by Mewton *et al*. and King *et al*., are two indices that combine global systolic performance with anatomic information^11,12^. They are derived from non-contrast cine CMR images and do not require additional post-processing software. In the present study, it was hypothesized that compared with conventional indices, LVGFI and MCF provide better evaluation of myocardial dysfunction and are better at discriminating CA from the other patients with thickened ventricular walls.

The purpose of this study was to evaluate LVGFI and MCF in their ability to discriminate CA from HCM without using contrast agents on CMR. To our knowledge, this is the first study that used LVGFI to aid in the diagnosis of thickened hearts.

## Results

### Demographic and clinical characteristics of the included patients

Demographic and clinical characteristics of 158 included patients and 35 normal controls are presented in Table 1. Patients with CA (n = 68) were older and had higher NYHA functional classes than HCM patients (n = 90). Further, 69.1% of patients with CA were classified as NYHA III-IV and 66.2% were light chain amyloidosis type. The HCM group had higher body mass index and greater percentage of females. Moreover, more hypertensive patients were found in this group than in the CA group. NT-proBNP and Troponin T levels were markedly elevated in both groups and were significantly higher in the CA group (log Troponin T: 4.6 ± 0.9 vs. 3.0 ± 0.9; log NT-proBNP: 8.6 ± 1.2 vs. 6.9 ± 1.0; *p* < 0.001 for both). Nine (11.7%) and seven (7.8%) patients in the CA and the hypertrophic control groups had a history of atrial fibrillation, respectively. Healthy subjects were slightly younger than the two groups of patients (ANOVA, *p* < 0.05).

**Table 1:** Demographic and clinical characteristics of the included subjects.

	CA (n = 68)	HCM (n = 90)	NC (n = 35)	P values
Age, years	59.7 ± 10.1*	53.0 ± 16	51.0 ± 9.0	0.001
Gender, females, n(%)	28 (41.1%)	36 (40%)	19 (54%)	ns
BMI, kg/m^2^	22.2 ± 3.4	24.7 ± 3.7	23.6 ± 2.3	ns
NYHA III-IV, n(%)	47 (69.1%)*	11 (12.2%)	—	<0.001
Hypertension, n(%)	10 (14.7%)*	27 (31.0%)	—	0.02
Diabetes, n(%)	4 (5.8%)	6 (6.9%)	—	ns
Sub-types of HCM	—		—	—
Asymmetrical (RWT > 1.3)/LOVOT		67/40		
Concentric (RWT < 1.3)		14		
Mid-ventricular		2		
Apical		7		
AL, n(%)	45 (66.2%)	—	—	—
Atrial fibrillation, n(%)	8 (11.7%)	7 (7.8%)	—	ns
NT-proBNP, pg/ml	5792 (3059.5, 11505)*	1244 (452, 1831.7)	—	<0.001
Troponin T, pg/ml	123 (70.5, 219.8)*	16.6 (11.1, 26.7)	—	<0.001
**Medications**				
Chemotherapy	18 (26.5%)	—	—	
Diuretics	45 (66.2%)	7 (7.8%)	—	
β-blockers	11 (16.2%)	31 (34.4%)	—	
ACEI/ARB	2 (3%)	8 (8.9%)	—	
Ca^2+^ blockers	2 (3%)	11 (12.2)	—	
Statin	4 (6%)	8 (8.9%)	—	

CA, cardiac amyloidosis; HCM, hypertrophic cardiomyopathy; NC, normal controls; NYHA, New York functional classification; LVOTO, left ventricular outflow tract obstruction; AL, light-chain amyloidosis; ns, not significant; ACEI, angiotensin-converting enzyme inhibitor; ARB, angiotensin receptor blocker.

### CMR characteristics of CA and HCM patients

**Figure 1:**
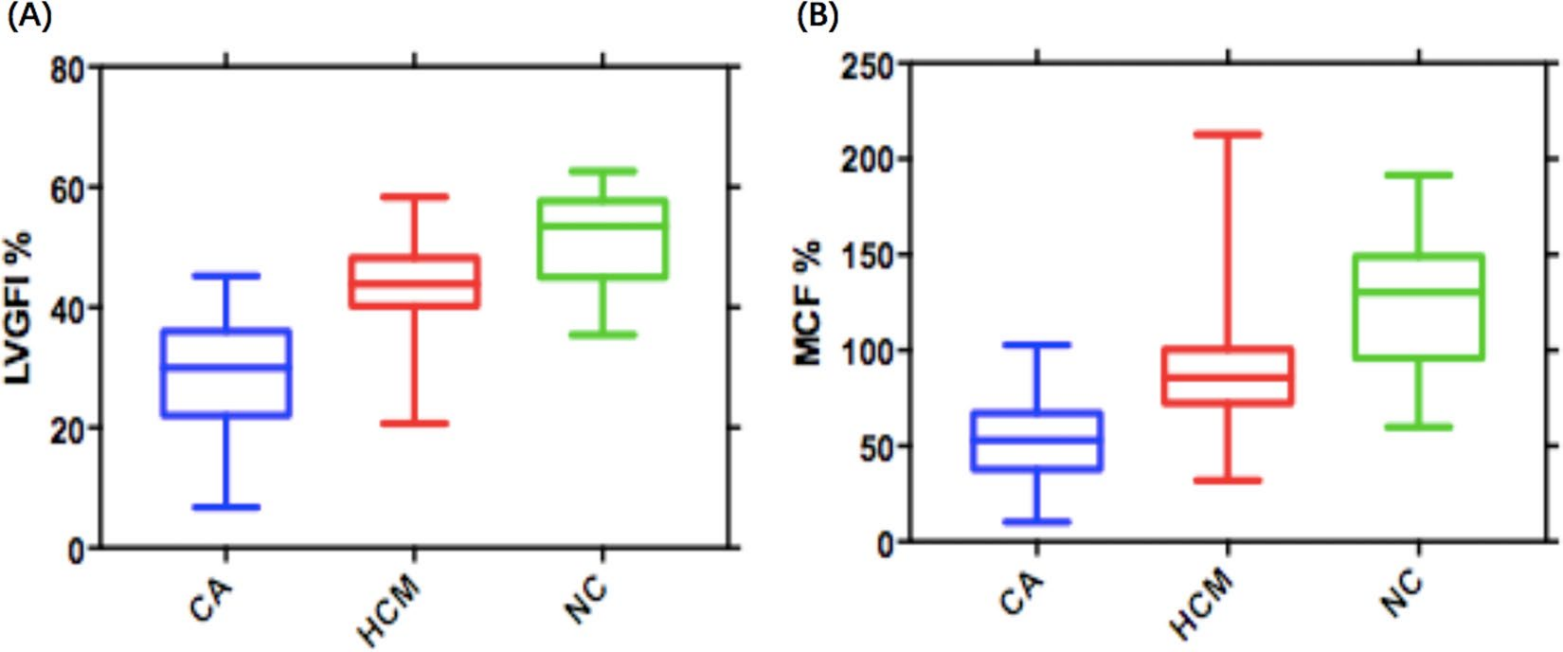
Comparisons of left ventricular global index (LVGFI) and myocardial contraction fraction (MCF) among cardiac amyloidosis (CA), hypertrophic cardiomyopathy (HCM) and normal controls (NC).

LV morphological and functional parameters are presented in Table 2. The maximum wall thickness (mWT) and LV mass were larger in the two patient groups than in healthy controls. LV mass was comparable in the two patient groups (*p* = 0.64), whereas mWT was higher in the HCM group than in CA (*p* = 0.01). LVGFI and MCF were significantly higher in healthy controls (Fig. 1). LVEF was comparable between HCM patients and healthy subjects (*p* = 0.91). And 58.8% CA patients had preserved LVEF (LVEF ≥ 50%). CA patients displayed remarkably higher LGE extent than HCM (p < 0.001). LVGFI and MCF were correlated with LGE extent (LVGFI: r = −0.516, MCF: r = −0.421; *p* < 0.001). Furthermore, Correlation analyses revealed that LVGFI and MCF also had strong associations with troponin T (LVGFI: r = −0.640, MCF: r = −0.566; *p* < 0.001) and NT-proBNP levels (LVGFI: r = −0.601, MCF: r = −0.526; *p* < 0.001).

**Table 2:** LV functional and morphological parameters in patients and healthy subjects.

	CA (n = 68)	HCM (n = 90)	NC (n = 35)	*P* values (CA vs HCM)
mWT (mm)	16.8 (14.5, 19.0)*^,†^	18.5 (15.1, 22)*	8.3 ± 1.1	0.01
Mass	108.4 (87.2, 133.4)*	116.8 (88.3, 138.7)*	66.4 ± 17.2	0.65
EDV (ml)	113.3 (95.8, 127.2)^†^	145.7 (118.3, 169.5)*	118.4 ± 25.4	<0.001
ESV (ml)	54.1 (43.4, 71.2)*	51.7 (42.1, 63.7)*	43.8 ± 10.2	0.29
SV (ml)	54.7 (42.0, 64.3)*	88.2 (73.2, 107.4)*	74.4 ± 16.6	<0.001
LVEF (%)	51.0 (40.7, 57.9)*^,†^	62.9 (59.7, 66.9)	62.9 ± 4.0	<0.001
MCF (%)	53.1 (38.1, 66.4)*^,†^	86.5 (72.6, 100.2)*	123.2 ± 30.7	<0.001
LVGFI (%)	30.0 (22.1, 35.9)*^,†^	43.9 (40.4, 48.1)*	51.9 ± 7.3	<0.001
Quantitative LGE mass (g)	59.5 ± 4.2	18.8 ± 2.0	—	<0.001

*P < 0.05 between healthy subjects and patients. ^†^*P* < 0.05 between CA and HCM. CA, cardiac amyloidosis; HCM, hypertrophic cardiomyopathy; NC, normal controls; mWT, maximum wall thickness; EDV, end-diastolic volume; ESV, end-systolic volume; SV, stroke volume; MCF, myocardial contraction fraction; LVGFI, left ventricular global function index; LGE, late gadolinium enhancement.

### Diagnostic performance of CMR parameters

**Figure 2:**
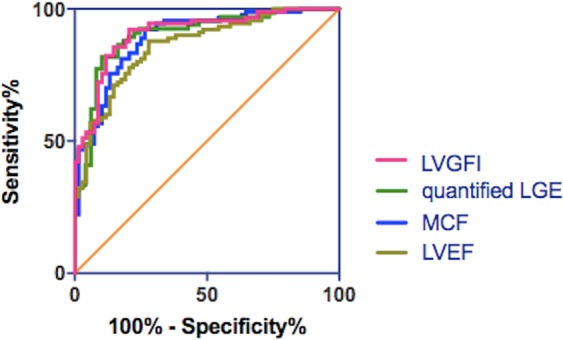
Receiver operating characteristic curves for left ventricular global index (LVGFI), myocardial contraction fraction (MCF), quantified LGE and left ventricular ejection fraction (LVEF) in differentiating cardiac amyloidosis (CA) from hypertrophic cardiomyopathy (HCM).

ROC analyses for discriminating the two patient groups from the normal subjects is presented in supplementary Table 1. The differential diagnostic performance of conventional and novel CMR parameters for CA is displayed in Table 3 and Fig. 2. Significance of differences among the AUCs of these parameters was verified by Delong’s test (LVGFI vs LVEF, *p* = 0.011; MCF vs LVEF, *p* = 0.256; LVGFI vs quantified LGE, *p* = 0.68). Multiple logistic regression analysis revealed that LVGFI (OR: 1.11, 95% CI: 1.01–1.23; *p* = 0.034), LGE (OR: 0.90, 95% CI: 0.86–0.95, *p* < 0.001), log NT-proBNP (OR: 0.42, 95% CI: 0.19–0.96, *p* = 0.039) and log Troponin (OR: 0.36, 95% CI: 0.14–0.89, *p* = 0.028) were independent predictors of CA.

**Table 3:** Discriminatory capacity of the CMR parameters in differentiating CA from HCM.

Parameters	AUC	95% CI	Cutoff value	Sen%	Spe%
LVGFI	0.91	0.86–0.96	37.4	87.8	80.9
MCF	0.89	0.84–0.94	70.4	81.1	82.3
LVEF	0.86	0.80–0.91	58.5	77.8	79.4
LVEF + LV mass	0.86	0.80–0.91	—	—	—
Quantitative LGE mass (g)	0.90	0.84–0.96	28.5	81.8	89.8

CA, cardiac amyloidosis; HCM, hypertrophic cardiomyopathy; LVGFI, left ventricular global function index; MCF, myocardial contraction fraction; LGE, late gadolinium enhancement; AUC, area under the receiver operating characteristic curve; Sen, sensitivity; Spe, specificity.

### Diagnostic values of parameters in patients with preserved LVEF and similar hypertrophy

**Figure 3:**
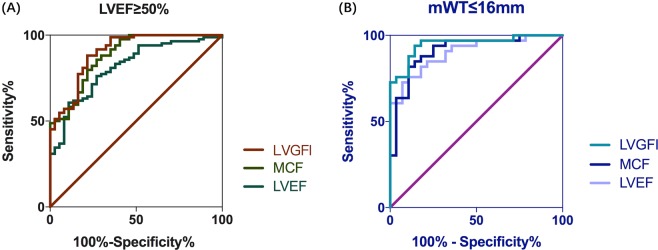
Receiver operating characteristic curves for left ventricular global index (LVGFI), myocardial contraction fraction (MCF) and left ventricular ejection fraction (LVEF) in differentiating cardiac amyloidosis (CA) from hypertrophic cardiomyopathy (HCM) in patients with preserved LVEF (**A**) and similar hypertrophy (**B**).

Diagnostic values of LVGFI and MCF in patients with similar hypertrophy and preserved LVEF (≥50%) are presented in Table 4 and Fig. 3. In patients with similar hypertrophy, LVGFI demonstrated great performance (AUC = 0.92, 95% CI [0.87–0.97]) with both high sensitivity (91.1%) and specificity (81.0%). For the preserved LVEF subgroup, LVGFI also had a high AUC (0.90, 95% CI [0.84–0.96]), with great sensitivity (88.1%) and moderate specificity (78.4%).

**Table 4:** Diagnostic values of parameters in subgroups with preserved LVEF and similar hypertrophy.

	HCM vs CA with preserved LVEF (CA = 37, HCM = 84)					*P* value (against LVEF)
	AUC	95% CI	Cutoff	Sen (%)	Spe (%)	
LVGFI	0.90	0.84–0.96	39.1	88.1	78.4	0.01
MCF	0.88	0.82–0.95	70	85.7	73.0	0.11
LVEF	0.81	0.73–0.89	60.7	76.2	73.0	—
	**CA vs HCM with similar hypertrophy (CA = 68, HCM = 56)**					***P*** **value (against LVEF)**
	**AUC**	**95% CI**	**Cutoff**	**Sen (%)**	**Spe (%)**	
LVGFI	0.92	0.87–0.97	37.4	91.1	81.0	0.01
MCF	0.91	0.86–0.96	64.9	94.6	73.5	0.19
LVEF	0.86	0.81–0.92	55.0	89.3	70.6	—

CA, cardiac amyloidosis; HCM, hypertrophic cardiomyopathy; MCF, myocardial contraction fraction; LVGFI, left ventricular global function index; AUC, area under the receiver operating characteristic curve; Sen, sensitivity; Spe, specificity.

### Reproducibility

Twenty subjects were reanalyzed by a second investigator who was blinded to clinical information to assess the interobserver agreement. The results of all the included parameters are shown in Table 5.

**Table 5:** Inter-observer variability of the included parameters.

	EDV	ESV	LV mass	LVEF	LVGFI	MCF
ICC	0.991	0.988	0.988	0.950	0.945	0.924
95% CI	0.978–0.996	0.971–0.985	0.972–0.995	0.881–0.980	0.862–0.978	0.819–0.968

ICC, intraclass correlation coefficient.

## Discussion

The major findings of the present study were as follows: (1) LVGFI and MCF are two novel parameters that could determine myocardial function in ventricular hypertrophy, (2) LVGFI was better than LVEF, and comparable to quantified LGE at differentiating CA from HCM; (3) LVGFI still demonstrated outperformance under difficult diagnostic conditions of similar hypertrophy or preserved LVEF.

MCF was initially described by King *et al*. as a three-dimensional, volumetric measure of myocardial shortening and was used to distinguish between patients with hypertensive heart disease (HHD) and athletes with physiologic hypertrophy^12^. From a mathematic perspective, SV is a measure of the difference between end-diastolic and end-systolic volumes that determine the output of blood from each myocardial contraction. Because it is the myocardium that contracts during systole, by dividing SV by myocardial volume, MCF represents an index of myocardial contraction that is not influenced by volume. Arenja *et al*.^13^ assessed MCF in patients with heart failure and LVH and showed that MCF outperformed LVEF and LVMI in discriminating CA from other forms of LVH. In the present study, MCF was significantly reduced in CA and HCM groups for different reasons. Decreased MCF in patients with CA is a result of decrease in SV and increase in myocardial volume. In early stage HCM patients, SV remains normal or even larger, while myocardial volume increases in a larger scale, thus resulting a decreased MCF. However, MCF was not statistically superior to LVEF at differentiating CA from HCM in our study group.

The present study showed that LVGFI was superior to conventional LVEF in assessing and discriminating CA patients, and comparable to quantified LGE in the differential diagnosis. As described by Mewton *et al*., LVGFI represents the relationship between SV and total heart size, including myocardial volume and mean LV cavity volume (mean of end-diastolic and end-systolic volumes)^11^, which carries additional information about cardiac remodeling. As it was reported by previous studies, LVGFI could provide incremental prognostic value in the prediction of major adverse cardiac events in patients ﻿after ST-segment elevation myocardial infarction^14,15^. In the present study, LVGFI decreased in both CA and HCM groups. And LVGFI had strong correlation with myocardial fibrosis assessed by LGE. In the HCM group, LV cavity volume is reduced because of increased wall thickness, and to preserve SV, the end-diastolic volume is enlarged through remodeling, which is consistent with findings of the present study. EDV was not significantly increased in CA, probably because amyloid deposition in the myocardium has caused more pronounced diastolic dysfunction. Different pathophysiologic mechanisms that have led to alterations in LV function and structure are comprehensively expressed by LVGFI. And this might be the foremost reason that LVGFI could outperformed conventional functional parameter in differentiating CA from HCM. And we had also found that LVGFI had comparable diagnostic capacity to LGE, which had been widely used in clinical settings.

In patients with similar hypertrophy, LVGFI continued to be an accurate parameter with high sensitivity and specificity. In this condition, myocardial volume of the two group had no significant difference. But amyloid deposition could cause a thickened and stiffened ventricular wall, restraining EDV to dilate, leading to a dramatically decreased LVGFI. On the contrary, early stage HCM could preserve cardiac output by enlarging EDV. So decreased LVGFI in HCM would not be as reduced as CA. In the presence of preserved LVEF, LVGFI still demonstrated excellent AUC with high sensitivity and moderate specificity. Differentiating between patients with similar hypertrophy and preserved LVEF are difficult issues that clinicians may frequently encounter. Subgroup analyses in the present study indicated that LVGFI can be powerful even in these challenging clinical settings.

The formulas for calculating LVGFI and MCF are similar. They both account for the relationship between LV mass and LV dimensions. However, LVGFI accounts for alterations in LV mass and overall LV cavity size that reflect remodeling in the heart, which demonstrates intrinsic pathophysiologic characteristics of the amyloid deposits-hypertrophy. We also found that the superiority of LVGFI was not a result of a simple combination of basic LV geometric measurements, such as ESV, EDV and LV mass. When we use a linear combination of LVEF and LV mass to differentiate CA from HCM, the diagnostic value was the same as LVEF. And this is because there was no significant difference of LV mass between the two groups (p = 0.65).

Troponin T level was markedly elevated in hypertrophic patients in this study, which is consistent with previous studies^16,17^. The present study also demonstrated that LVGFI and MCF are strongly associated with troponin T and NT-proBNP. The CA group had significant higher levels of troponin T, NT-proBNP, and NYHA than HCM in this study mainly due to the following reasons. For one thing, cardiac amyloidosis is a more aggressive disease with poor prognosis. Amyloid deposits can cause myocardial microvascular dysfunction, decrease in LV systolic function, and cardiac troponin release^18^. For another, circulating amyloid light chain seems to have a direct cardiotoxic effect, which could impair the cardiac function. Last but not least, the atrial chamber would be dilated as a result of the more pronounced diastolic dysfunction in CA patients, which could lead to a great release of natriuretic peptide.

The retrospective nature of this study carries its major limitations. The lack of cardiac biopsy and genetic testing in these patients made it impossible to precisely differentiate the subtypes (AL, ATTRv, and ATTRwt) of cardiac amyloidosis. In addition, sub-analysis of patients with preserved LVEF was limited by small sample size. Further well-designed prospective studies are needed to determine the utility of these two parameters for a more general application.

## Conclusions

LVGFI is a novel and clinically useful parameters with excellent ability in determining myocardial function and differentiating cardiac amyloidosis from hypertrophic cardiomyopathy.

## Methods

### Patient population

All the patients with increased ventricular wall thickness who underwent CMR between 2015 and 2018 at West China Hospital were retrospectively reviewed.

CA was diagnosed on the basis of myocardial biopsy, if performed. If not, cardiac involvement of amyloidosis was considered if mean LV wall thickness (septum and posterior wall)≥12 mm on echocardiography (EEG) or CMR in conjunction with a positive extra-cardiac biopsy and conditions such as plasma cell dyscrasia, and multiple myeloma^19^. The HCM group was included according to the 2014 ESC Guidelines on diagnosis and management of hypertrophic cardiomyopathy^20^.

Exclusion criteria included patients with valvular heart disease (greater than mild stenosis or greater than moderate regurgitation), significant coronary artery disease, and other confirmed systemic diseases. Hypertensive patients with concentric hypertrophy were also excluded.

We also included a normal control group, which was confirmed by no evidence of structural or functional heart diseases on CMR, no clinical heart disease history and no electrocardiogram abnormalities. The New York Heart Association Classification (NYHA) was performed by the patient’s referring cardiologist. Baseline characteristics and laboratory biomarkers were assessed within one week of the CMR examinations. This study was approved by the Institutional Ethics Review Board of West China Hospital and in accordance with the Declaration of Helsinki. All participants signed written informed consent before the CMR scans.

### CMR protocol

CMR scans were acquired using standard institutional CMR protocols with a 3.0 Tesla MR system (MAGNETOM Skyra, Siemens Healthcare, Erlangen, Germany). Cine images were obtained using balanced steady-state free-precession sequences in consecutive short axis covering the LV (8–12 slices from the level of the mitral valve annulus to the LV apex) and in the long axis (two-, three-, and four-chamber views). Scanning parameters were as follows: repetition time (TR), 39.34 ms and echo time (TE) 1.22 ms; flip angle, 38°; field of view, 284 × 399 mm; matrix size 139 × 208 mm; and slice thickness, 8 mm. 25 cardiac phases were retrospectively reconstructed for each slice level. LV volumes, and wall thickness were all assessed from these continuous cine sequences. Late gadolinium enhancement (LGE) imaging was acquired 10–15 min after contrast injection by using an ﻿inversion recovery TrueFISP sequence (TR/TE = 870.4/1.18 ms; flip angle = 40°; slice thickness = 8 mm; field of view = 400 × 275 mm^2^; matrix size = 176 × 256).

### CMR analysis

CMR analysis was conducted using a commercially available software (CVI^42^; Circle Cardiovascular Imaging, Inc., Calgary, Canada). Endocardial and epicardial contours were manually delineated by two experienced radiologists in consecutive short-axis slices at end-diastolic and end-systolic phases. The radiologists were blinded to the patients’ clinical profiles. Trabecular tissue and papillary muscles were excluded from LV mass. LGE extent was quantified by 5 standard deviations (SD) above the signal intensity of remote normal myocardium^21^.

To further investigate the diagnostic performance of LVGFI and MCF in more challenging conditions, two subgroup analyses were conducted in patients with similar hypertrophy and preserved LVEF (LVEF ≥ 50%).

### Assessments of LVGFI and MCF

LVGFI was calculated according to equation (1) and expressed as a percentage^11^. LV global volume was defined as the sum of the mean LV cavity volume [(LVEDV + LVESV)/2] and the myocardial volume. LV myocardial volume was calculated as LV myocardial mass divided by myocardial density, which is specified as 1.05 g/mL^22^.

1 $${LVGFI}=\frac{{EDV}-{ESV}}{\frac{{EDV}+{ESV}}{2}+{LV}\,{mass}/1.05}\times 100 \% $$

MCF was calculated by dividing LVSV by LV myocardial volume^12^.

2 $${MCF}=\frac{{EDV}-{ESV}}{{LV}\,{mass}/1.05}\times 100 \% $$

The formula of left ventricular ejection fraction:

3 $${LVEF}=\frac{{EDV}-{ESV}}{{EDV}}\times 100 \% $$

### Statistical analysis

Data were expressed as mean ± standard deviation or median (interquartile range). Continuous variables were tested for normal distribution using Kolmogorov–Smirnov test. CMR measures were compared between CA and the control group using independent samples t-test or Mann–Whitney rank sum test, as appropriate. Comparisons between multiple groups were conducted using one-way analysis of variance or Kruskal–Wallis test with post hoc Bonferroni correction. Receiver operating characteristic (ROC) curve analysis was conducted to delineate the diagnostic accuracy of LVGFI and MCF. Areas under the curves (AUC) were statistically compared. NT-proBNP and Troponin T were log-transformed because of their skewed distribution. Correlations were assessed using Pearson’s correlation coefficient. To assess whether LVEF, LVGFI, MCF, LGE, log NT-proBNP, log Troponin T, and age had additional predictive values for the detection of amyloid myocardial infiltration, a forward and backward logistic regression with multiple covariates was performed using the diagnosis of amyloidosis as the outcome variable. *P* < 0.05 was considered statistically significant. Statistical analyses were conducted using SPSS (Version 19; IBM, Armonk, NY) and GraphPad Prism 7 (GraphPad, San Diego, Calif). Significance of the difference between the areas under ROC curves was verified using Stata (Version 13.0; Texas, USA).

## Supplementary information

Supplementary information

## Data Availability

The datasets used during the current study are available from the corresponding author on reasonable request.
