# Supplementary material for: Left ventricular global function index by magnetic resonance imaging — a novel marker for differentiating cardiac amyloidosis from hypertrophic cardiomyopathy
Source: Sci Rep. 2020 Mar 13;10:4707. doi: 10.1038/s41598-020-61608-9 (PMC7069935; doi:10.1038/s41598-020-61608-9)
Supplement: Supplementary file 1 — Supplementary information [file 41598_2020_61608_MOESM1_ESM.docx]

**Left ventricular global function index by magnetic resonance imaging — a novel marker for differentiating cardiac amyloidosis from hypertrophic cardiomyopathy**

Shan Huang, MS • Hua-yan Xu, MD • Kai-yue Diao, MD • Ke Shi, MD • Yong He, MD • Sen He, MD • Yi Zhang, MD • Yue Gao, MD • Meng-ting Shen, MS • Ying-kun Guo, MD • Zhi-gang Yang, MD

**Supplementary materials**

**Table 1. Outcome of ROC analyses for discriminating cardiac amyloidosis and hypertrophic cardiomyopathy from normal controls.**

|  | **AUC** | **95% CI** | **Cutoff** | **Sen (%)** | **Spe (%)** |
| --- | --- | --- | --- | --- | --- |
| **HCM (n=90) vs NC (n=35)** | | | | | |
| LVGFI | 0.78 | 0.69-0.88 | 51.4 | 60 | 87.8 |
| MCF | 0.82 | 0.73-0.90 | 115.8 | 62.9 | 88.9 |
| LVEF | 0.50 | 0.40-0.61 | 58 | 91.4 | 21.1 |
| **CA(n=68) vs NC (n=35)** | | | | | |
| LVGFI | 0.98 | 0.95-1.00 | 40.9 | 94.3 | 91.2 |
| MCF | 0.98 | 0.95-1.00 | 76.9 | 97.1 | 89.7 |
| LVEF | 0.90 | 0.85-0.95 | 58 | 91.4 | 76.4 |

CA, cardiac amyloidosis; HCM, hypertrophic cardiomyopathy; NC, normal controls; LVGFI, left ventricular global function index; MCF, myocardial contraction fraction; AUC, area under the receiver operating characteristic curve; Sen, sensitivity; Spe, specificity.

**Table 2. Comparisons of diastolic strain parameters between CA and HCM.**

| **Diastolic strain rates** | **CA** | **HCM** | **P value** |
| --- | --- | --- | --- |
| **dGRSR** | 0.8 (0.5, 1.1) | 1.2 (1.0, 1.6) | <0.001 |
| **dGCSR** | 0.6 (0.4, 0.8) | 0.8 (0.6, 1.0) | <0.001 |
| **dGLSR** | 0.5 (0.4, 0.6) | 0.6 (0.5, 0.7) | <0.001 |

dGRSR, diastolic global radial strain rate; dGCSR, diastolic global cirumferential strain rate; dGLSR, diastolic global longitudinal strain rate; CA, cardiac amyloidosis; HCM, hypertrophic cardiomyopathy.

**Figure 1. ROC analyses of diastolic strain rates in differentiating CA from HCM.**


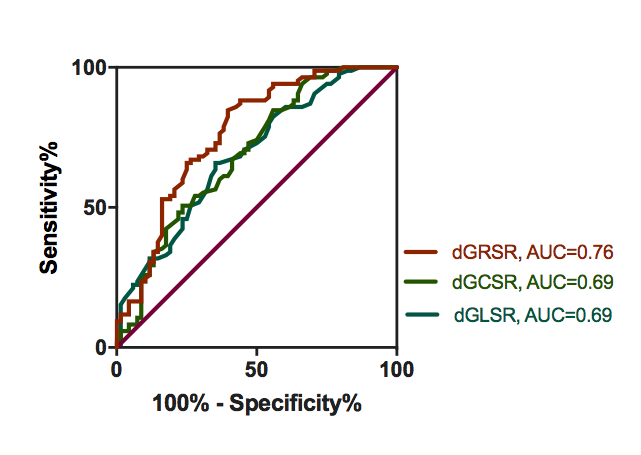


dGRSR, diastolic global radial strain rate; dGCSR, diastolic global cirumferential strain rate; dGLSR, diastolic global longitudinal strain rate; AUC, area under the receiver operating characteristic curve; CA, cardiac amyloidosis; HCM, hypertrophic cardiomyopathy.
